# METTL3-mediated m^6^A modification regulates cell cycle progression of dental pulp stem cells

**DOI:** 10.1186/s13287-021-02223-x

**Published:** 2021-03-01

**Authors:** Haiyun Luo, Wenjing Liu, Yanli Zhang, Yeqing Yang, Xiao Jiang, Shiqing Wu, Longquan Shao

**Affiliations:** 1 Shunde Hospital, Southern Medical University (The First People’s Hospital of Shunde), Foshan, 528308 China; 2 Stomatological Hospital, Southern Medical University, Guangzhou, 510280 China

**Keywords:** Dental pulp, Adult stem cells, RNA epigenetics, Cell apoptosis, Cell cycle

## Abstract

**Background:**

Dental pulp stem cells (DPSCs) are a promising cell source in endodontic regeneration and tissue engineering with limited self-renewal and pluripotency capacity. *N*^6^-methyladenosine (m^6^A) is the most prevalent, reversible internal modification in RNAs associated with stem cell fate determination. In this study, we aim to explore the biological effect of m^6^A methylation in DPSCs.

**Methods:**

m^6^A immunoprecipitation with deep sequencing (m^6^A RIP-seq) demonstrated the features of m^6^A modifications in DPSC transcriptome. Lentiviral vectors were constructed to knockdown or overexpress methyltransferase like 3 (METTL3). Cell morphology, viability, senescence, and apoptosis were analyzed by β-galactosidase, TUNEL staining, and flow cytometry. Bioinformatic analysis combing m^6^A RIP and shMETTL3 RNA-seq functionally enriched overlapped genes and screened target of METTL3. Cell cycle distributions were assayed by flow cytometry, and m^6^A RIP-qPCR was used to confirm METTL3-mediated m^6^A methylation.

**Results:**

Here, m^6^A peak distribution, binding area, and motif in DPSCs were first revealed by m^6^A RIP-seq. We also found a relatively high expression level of METTL3 in immature DPSCs with superior regenerative potential and METTL3 knockdown induced cell apoptosis and senescence. A conjoint analysis of m^6^A RIP and RNA sequencing showed METTL3 depletion associated with cell cycle, mitosis, and alteration of METTL3 resulted in cell cycle arrest. Furthermore, the protein interaction network of differentially expressed genes identified Polo-like kinase 1 (PLK1), a critical cycle modulator, as the target of METTL3-mediated m^6^A methylation in DPSCs.

**Conclusions:**

These results revealed m^6^A methylated hallmarks in DPSCs and a regulatory role of METTL3 in cell cycle control. Our study shed light on therapeutic approaches in vital pulp therapy and served new insight into stem cell-based tissue engineering.

**Supplementary Information:**

The online version contains supplementary material available at 10.1186/s13287-021-02223-x.

## Background

Adult stem cells residing in various tissues play an essential role in maintaining tissue homeostasis and ensuring regenerative needs. These cells may remain quiescent in a specialized microenvironment for a quite long time and can still differentiate into specific cell types once there are physical damages or activating signals [1]. The regenerative and self-renewal capacity of stem cells highlight the therapeutic approaches not only as regenerative medicine in local injury repair but also in tissue engineering. Adult stem cells with diverse biological properties had been reported in lots of tissues including the bone marrow, blood, muscle, skin, liver, and also teeth. Among these, the dental pulp is rich in mesenchymal stem cells which can be easily obtained from permanent and primary teeth as cell resources. Dental pulp stem cells (DPSCs) derived from cranial neural crest cells are responsible for dentine-pulp complex regeneration and possess superior self-renewal and pluripotency capacity than other adult stem cells [2–4]. DPSCs can continuously grow up to thirty passages and still maintain pluripotent to differentiate into odontogenic, chondrogenic, adipogenic, and neurogenic cells [3].

As other adult stem cells, DPSCs are capable to repair mild injury while fail to cope with irreversible or severe damage due to its limited regenerative potential. The long lifespan and persistence of adult stem cells contributed to the accumulation of multiple damages which resulted in regenerative capacity reduction and insufficient injury repair [5]. The reprogramming and self-renewal ability of adult stem cells peaked in immature states from young individuals, then declined along with age in older ones, and these results are consistent with clinical observation. In the dental pulp, researches had demonstrated a better success rate of vital-pulp preservation by indirect and direct pulp capping in young age especially immature teeth with incomplete root development [6, 7]. DPSCs of immature teeth exhibited superior self-renewal and regenerative potential; however, the stemness may gradually reduce over time in mature ones [8, 9]. Exploring the regulatory network and molecular mechanisms of DPSCs’ biological activities will greatly advance therapeutic approaches in vital pulp therapy and also stem cell-based tissue engineering.

*N*^6^-methyladenosine (m^6^A) is the most prevalent internal modification of messenger RNA and non-coding RNA in eukaryotes [10, 11]. Though m^6^A modification has been identified for decades, the contributions remained a mystery; until recently, a flurry of studies revealed its functional effects on gene expressions and diverse biological processes [11]. Reversible m^6^A modification can dynamically regulate almost every step in RNA metabolisms including mRNA stability, translation, non-coding RNA processing, and alternative splicing according to signal cues, and participate in multiple biological processes as homeostasis maintenance, stem cell fate determination, and also embryonic development [12–14]. In mammals, m^6^A marks are catalyzed by methyltransferase complex consisted of enzymatic subunit methyltransferase like 3 (METTL3) together with METTL14 and Wilms tumor 1-associated protein (WTAP) and can be demethylases by fat mass and obesity-associated protein (FTO) and alkB homolog 5 (ALKBH5). As the main “writer,” METTL3-mediated m^6^A modification was reported to modulate stem cell pluripotency, self-renewal, sex determination, and meiosis initiation [15–17]. Until now, little was known about the potential effect of m^6^A methylation and the molecular mechanism in DPSCs.

In this study, we aim to figure out the potential involvement of m^6^A modification in dental pulp hemostasis and elucidate the underlining molecular mechanism. Our study first demonstrated the features and distribution of m^6^A hallmarks in DPSCs by m^6^A RIP-seq and uncover a regulatory role of m^6^A methyltransferase-METTL3 in cell senescence and apoptosis. Furthermore, we discovered METTL3 make an effect on cell cycle control via a m^6^A-dependent manner which provide new insights into the RNA epigenetic mechanism in DPSCs.

## Methods

### Ethics statement

This entire study was approved by the human research committee of Shunde Hospital, Southern Medical University (the First People’s Hospital of Shunde), Foshan, China. The Shunde Hospital Southern Medical University Medical Ethics Committee reviewed all protocols. All subjects were informed and performed according to the guidelines after written informed consents were obtained.

### Dental pulp stem cells culture and characterization

Healthy dental pulp tissues from three independent donors were gently removed from the permanent teeth, and DPSCs were cultured as described previously [18]. Briefly, pulp tissues were isolated, then digested in collagenase type I (3 mg/ml) for 1 h to obtain single-cell suspensions. Cells were seeded into a 6-cm dish and cultured with Dulbecco’s modified Eagle’s medium supplemented with 10% fetal bovine serum and 1% penicillin/streptomycin at 37 °C under 5% CO_2_ condition. The medium was changed every 2 days, and DPSCs at passages 2–4 were used for the following experiments.

Flow cytometry was performed to identify DPSC phenotypes by screening the surface markers against CD29, CD34, CD44, CD45, and CD90 (BD Biosciences). Meanwhile, DPSCs were induced to differentiate into odontoblasts or adipocytes by an odontogenic or adipogenic medium as described in previous studies [19, 20]. After 7 days of induction by odontogenic medium, cells were fixed and stained with alkaline phosphatase (ALP), while 14 days for alizarin red staining (ARS). For adipogenic induction, cells were subjected to oil red O staining after 3 weeks of culture. For chondrogenic differentiation, DPSCs were cultured in an induction medium (Invitrogen) for 21 days before subjected to alcian blue staining to detect the extracellular matrix of chondrocytes.

### m^6^A-methylated RNA immunoprecipitation sequencing

The m^6^A-methylated RNA immunoprecipitation sequencing (m^6^A RIP-Seq) was used to observe the characterizations of m^6^A modification in DPSCs. Total RNA was isolated with TRIzol Reagent and enriched by Oligo(dT)-attached magnetic beads. The purified m^6^A RIPed RNA fragments of DPSCs were collected with the Millipore kit according to the protocol; purified mRNA was then fragmentized into about 100 nt for sequencing using fragmentation buffer. The Magna ChIP Protein A/G Magnetic Beads were incubated with m^6^A antibody in immunoprecipitation buffer. RNA fragmentation was enriched by magnetic beads and subjected to deep sequencing on an Illumina Novaseq™ 6000 platform at the LC-BIO Bio-Tech Ltd. as described previously [21].

### Real-time polymerase chain reaction

Total RNA from DPSCs were isolated with an RNeasy Mini kit (Foregene Biotec) and then reverse-transcribed with RT reagent kit (Takara Biotechnology) as described previously to obtain complementary DNA (cDNA). Real-time quantitative PCR was performed with Advanced™ SYBR Green Super Mix (Bio-Rad) according to the standard protocol. Data were analyzed using the standard curve method and normalized to GAPDH mRNA levels. The primer sequences used in real-time PCR were summerized in supplementary table 1.

To investigate specific target genes of METTL3, the m^6^A enrichment of PLK1 in m^6^A-IP RNA and input RNA as described before was quantified with quantitative PCR analysis for m^6^A RIP-qPCR.

### Microarray data

The gene expression array GSE52853 was obtained from the GEO database. GSE64392 which was submitted by Tamaoki et al. analyzed the gene expression profiles in DPSCs from mature and immature human permanent teeth [8]. In this study, GEO2R was used to identify the expression level of m^6^A-related genes for further study.

### Lentiviral vectors construction and infection

Two independent biological replicates of shRNA lentiviral vectors were used in this article. Number#1, vectors encoding homo METTL3 gene and green fluorescent protein (GFP) were pursued from Santa Cruz Company; number#2 vectors encoding METTL3 and negative control were constructed by Shanghai Genechem Company. The overexpression lentiviral vectors of homo METTL3 gene and GFP were also obtained from Genechem Company.

### Western blot analysis

The protein of DPSCs was lysed with RIPA buffer and assayed by the BCA protein assay kit (Thermo Scientific). Ten to thirty μg of protein in each group was subjected to SDS polyacrylamide gel electrophoresis and then transferred to polyvinylidene fluoride membranes (Millipore). The transferred proteins were reacted with primary antibody overnight at 4 °C and then labeled with secondary antibody for 1 h at room temperature. Primary antibodies in this study include METTL3 (1:1000; Cell Signaling Technology), GAPDH (1:2000; Proteintech Group), phosphorylation-p53 and p53 (1:1000; Santa Cruz), and PLK1 (1:1000; Cell Signaling Technology). The protein complexes were visualized with Super Signal-enhanced (Thermo Scientific) chemiluminescence.

### β-Galactosidase staining

DPSCs transfected with shMETTL3 or shCTR lentivirus were fixed and washed with PBS before β-galactosidase (β-gal) staining to detect cell senescence [22]. Cells were subjected to staining solution for 16–24 h at 37 °C according to the β-gal staining kit (Beyotime Biotechnology). Six randomly chosen images were obtained, and then β-gal-positive cells were counted.

### Flow cytometric analysis

DPSCs transfected with shRNA or overexpression lentivirus were plated in a 6-well plate. Then, cells were subjected to APC-conjugated Annexin V/propidium iodide assay (KeyGEN Biotechnology) according to a previous study and analyzed using flow cytometry [23].

### TUNEL assay

Apoptosis was also evaluated with the TUNEL Apoptosis Assay Kit (KeyGEN Biotechnology). Briefly, DPSCs transfected with shRNA lentivirus were fixed and stained with TUNEL reaction mixture as protocol. Six randomly fluorescence images were obtained, and percentage of red-labeled TUNEL-positive cell was analyzed by ImageJ.

### RNA sequencing and profile analysis

For sequencing analysis, total RNA was extracted by TRIzol Reagent and purified with Oligo(dT)-attached magnetic beads. The purified RNA fragments were converted into double-stranded cDNA. Paired-end runs with 250–350 base pair (bp) read lengths were used for RNA sequencing (RNA-seq) by Novaseq™ 6000 following the manufacturer’s recommendations. FastQC and FASTX toolkit were used to control data quality and map to homo reference genomes by HISAT2. Differentially expressed genes with fold change ≥2.0 and *p* value < 0.05 were subjected to analysis.

The enrichment of Gene Ontology (GO) was performed with R package GOseq and the DAVID database. Then, we mapped the differentially expressed genes to the Search Tool for Retrieval of Interacting Genes (STRING) database to obtain the protein-protein interactions, and the networks were displayed by Cytoscape 3.5.1.

### Cell cycle analysis

METTL3 knockdown and overexpression vectors were transduced in DPSCs, wihle PLK1 inhibitor BI2536 (1 nM, MedChemExpress) [24] or DMSO as negative control were used to inhibit PLK1 expression in shMETTL3-DPSCs. DPSCs were washed with PBS and then fixed in 70% cold ethanol overnight. At least 50,000 cells were subjected to propidium iodide staining with a Cell Cycle and Apoptosis Analysis Kit (Beyotime), and the DNA content was measured by fluorescence-activated cell sorting (FACS) instrument (BD Biosciences).

### Statistical analysis

Experiments were carried out at least three times, and the data was presented as mean ± standard deviation. Statistical significance (*p* < 0.05) level was determined by analyzing comparisons of variance using Graphpad Prism 7.0.

## Results

### Characterizations and m^6^A modification features of DPSCs

**Fig. 1:**
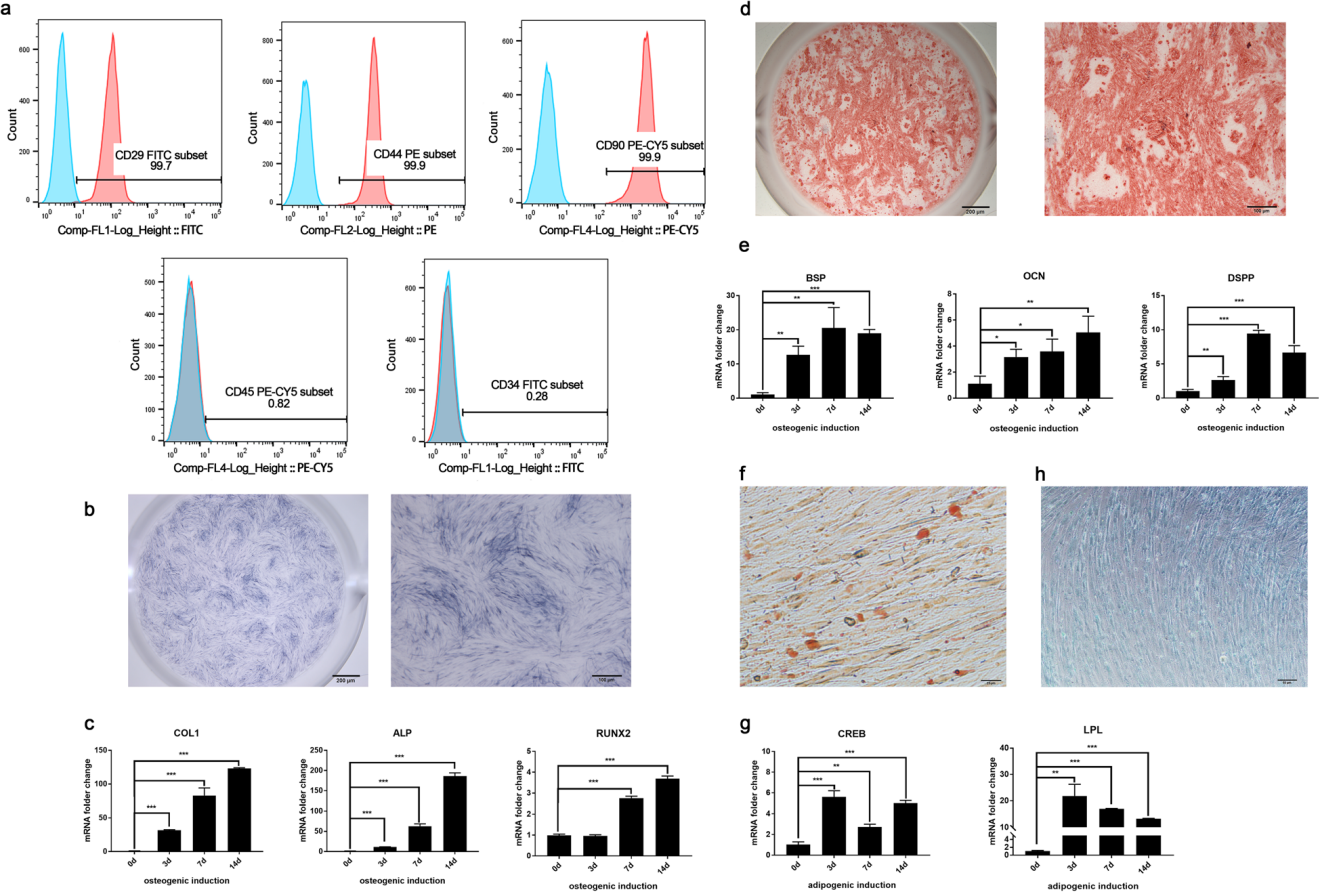
Characterizations and regenerative capacity of DPSCs. **a** Mesenchymal stem cell antigen (CD29, CD44, CD90) and hematopoietic cell antigen (CD34, CD45) expressed in DPSCs were detected by flow cytometry. **b** Alkaline phosphatase (ALP) straining of DPSCs after 7 days of odontogenic induction. **c** mRNA expression of early odontogenic markers COL1, ALP, and RUNX2 analyzed by PCR. **d** Calcium mineralization formation observed by alizarin red S staining (ARS) at day 14 in DPSCs. **e** mRNA expressions of late odontogenic genes-BSP, OCN, DSPP were assayed by PCR. **f** Accumulation of lipid vacuoles was detected by oil O staining. **g** mRNA expression of CREB and LPL after adipogenic induction detected by PCR. Significance was determined via Student’s *t* test analysis of variance (*n* = 3); data are represented as mean ± SD. *p < 0.05. **p < 0.01. ***p < 0.001

DPSCs appeared as spindle-like, elongated fibroblastic shape (Supplementary Figure S1). The surface markers of DPSCs were analyzed by flow cytometry; mesenchymal stem cell markers such as CD29, CD44, and CD90 were highly expressed with over 90% positive cells, while the expression of CD34 and CD45 (hematopoietic cell antigen) can be barely detected (Fig. 1a). After odontogenic medium induction for 3, 7, and 14 days, the early differentiation stage of DPSCs was evidenced by strong alkaline phosphatase (ALP) straining and upregulated osteo/odontogenesis-related markers such as RUNX2, ALP, and COL1 (Fig. 1b, c) while late differentiation stage identified by significant calcium mineralization in alizarin red staining (ARS) and mRNA expression of BSP, DSPP, and OCN (Fig. 1d, e). Meanwhile, accumulation of lipid vacuoles was detected by oil O staining in DPSCs after adipogenic induction, accompanying with increased expression of adipogenic-related markers as CREB, LPL (Fig. 1f, g). After chondrogenic differentiation, DPSCs were positive for alcian blue staining which indicated the glycosaminoglycan formation (Fig. 1h). These data suggested that DPSCs were capable of differentiating into odontoblasts and adipocytes which verified multiple differentiation potential and regenerative capacity did exist in DPSCs.

**Fig. 2:**
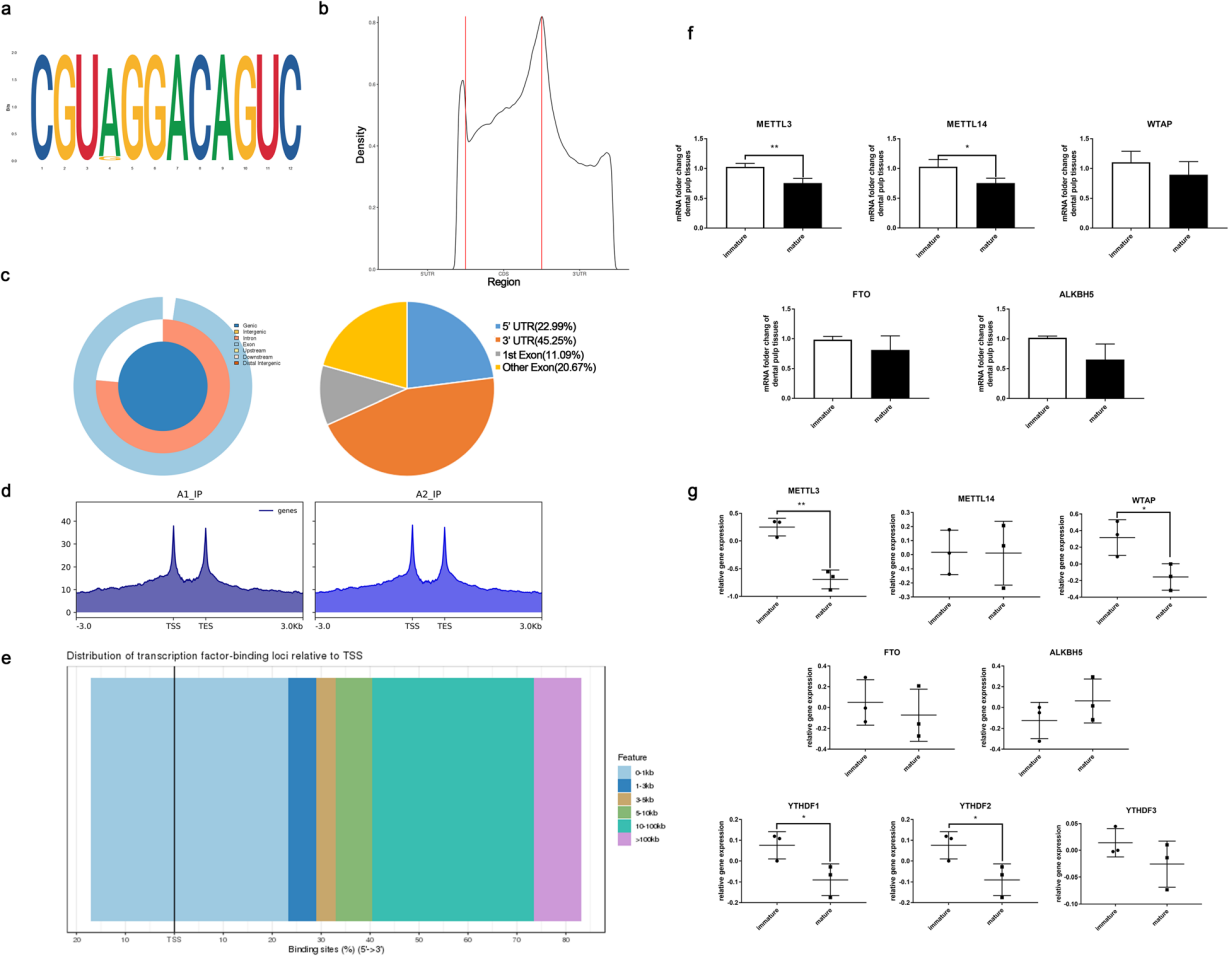
m^6^A modification features and related gene expressions in DPSCs. Expression profiles of m^6^A peak fraction and distribution in DPSCs transcript segments were analyzed by m^6^A RIP-seq. **a** Specific sequence motif of m^6^A peaks identified by the HOMER database. **b** Distribution of m^6^A peaks in 3′ UTR, CDS region, and 5′ UTR. **c** Pie chart analysis of m^6^A peak fraction in transcript segments. **d** The distribution of transcription factor-binding loci relative to transcription start sites (TSS). The m^6^A-related gene expressions in immature and mature dental pulp tissues and DPSCs. **e** mRNA expressions of METTL3, METTL14, WTAP and FTO, and ALKBH5 in immature dental pulp tissues and mature ones evaluated by PCR. **f** In the GEO databases, relative gene expressions of METTL3, METTL14, WTAP and FTO, ALKBH5, YTHDF1, YTHDF2, and YTHDF3 in immature DPSCs and immature ones were analyzed by GEO2r. Quantitative data are represented as mean ± SD, Student’s *t* test. *p < 0.05. **p < 0.01. ***p < 0.001

As the most prevalent RNA modification, m^6^A marks can dynamically regulate RNA metabolisms and participate in stem cell fate determination. To explore the binding area, motif, and distribution of m^6^A modifications in DPSCs, m^6^A immunoprecipitation with deep sequencing (m^6^A RIP-seq) was performed. We identified high similarity and overlapping of m^6^A peaks in two independent replicates and significantly enriched in GGm6ACA motif which is consistent with previous reports (Fig. 2a). A total of 13,191 m^6^A-modified transcripts were identified in m^6^A RIP-seq (*p* value < 0.05, fold enrichment> 1). The m^6^A peaks were abundant in 3′ untranslated regions (3′ UTR) 45.25%, exons 31.76%, 5′ UTR 22.99% (Fig. 2b, c), and transcription factor-binding sites were predominantly distributed within 100 kb related to transcription start sites (TSS) (Fig. 2d).

### METTL3 knockdown induced cell apoptosis and senescence of DPSCs

The dental pulp in immature teeth with incomplete root development preserved better regenerative potential than mature teeth [8]. We further investigated the gene expressions related to m^6^A modification including “writers” (METTL3, METTL14, WTAP), “erasers” (FTO, ALKBH5), and “readers” YTH domain-containing family protein (YTHDF1, YTHDF2, YTHDF3). The high expression levels of “writers” METTL3 and METTL14 in immature dental pulp tissues were observed compared with mature ones (Fig. 2e). In the global transcriptome profile of DPSCs in different developmental stages, the relative gene expressions of METTL3, WTAP, YTHDF1, and YTHDF2 were reduced in mature DPSCs compared to immature ones, while others had no significant differences (Fig. 2f).

**Fig. 3:**
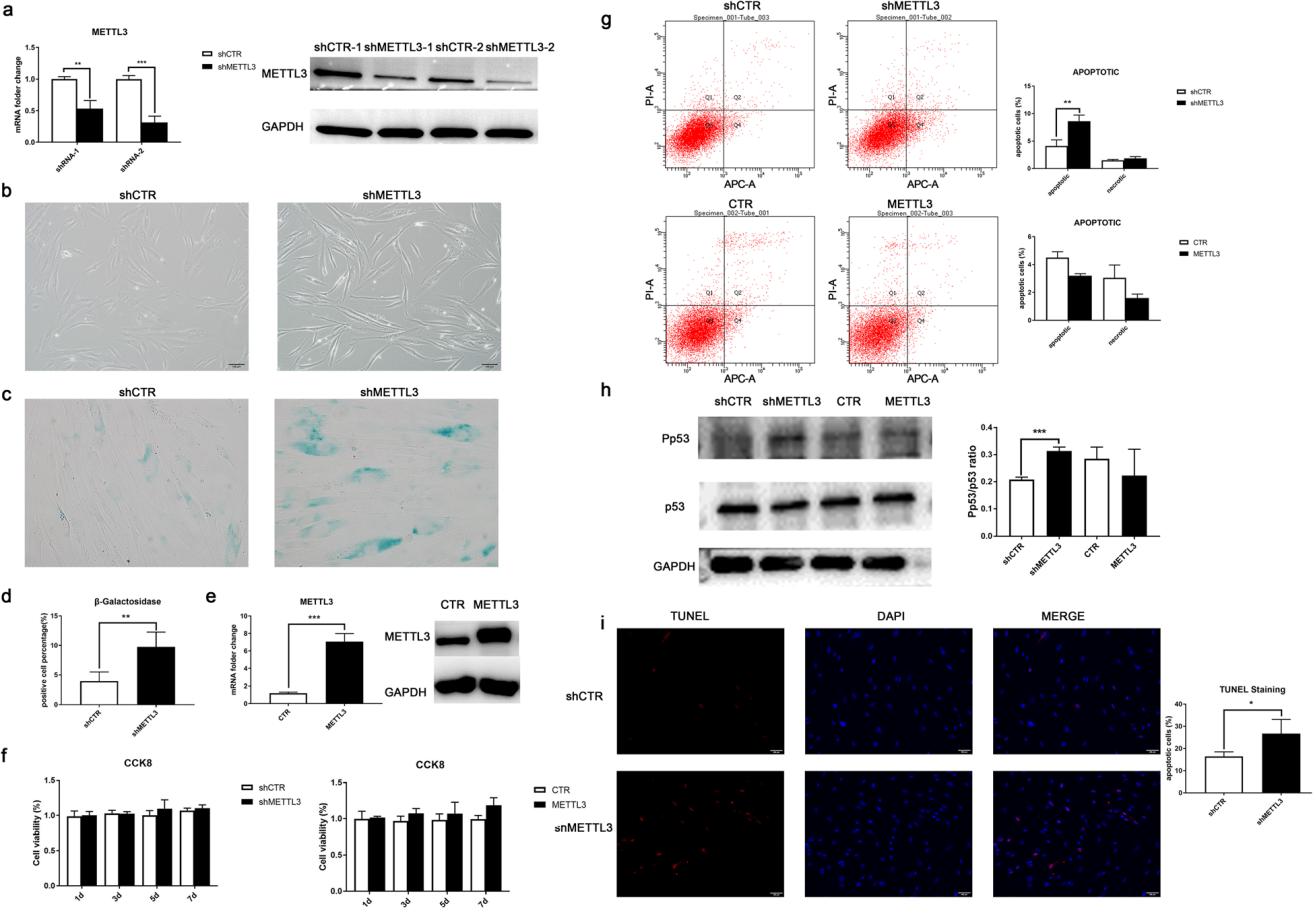
METTL3 knockdown induced cell apoptosis and senescence of DPSCs. **a** shRNA lentiviruses inhibited mRNA and protein expression of METTL3 confirmed by PCR and Western blot. **b** Representative images of DPSCs after METTL3 knockdown were captured with an inverted microscope. **c** β-Galactosidase staining was used to detect cell senescence in shCTR and shMETTL3-DPSCs. **d** Statistical analysis of positive β-galactosidase staining cells in green defined as senescence. **e** Lentiviral vector of METTL3 overexpression was transduced into DPSCs, and upregulated expression levels of mRNA and protein were confirmed by PCR and Western blot. **f** CCK8 assay analyzed cell viability of METTL3 knockdown and overexpressed DPSCs. **g** Flow cytometry of Annexin V/PI staining was employed to analyze the apoptotic rate after METTL3 inhibition and overexpression. **h** Phosphorylation-p53(Pp53) and p53 in shMETTL3-DPSCs were assayed by Western blot and evaluated by ImageJ. **i** TUNEL assay analyzed DPSC apoptosis induced by METTL3 knockdown. Quantitative data are represented as mean ± SD (*n* ≥ 3), Student’s *t* test. *p < 0.05. **p < 0.01. ***p < 0.001. shMETTL3-1 and METTL3-2 are 2 independent shRNAs used to knockdown METTL3 expression, and shCTR as negative control shRNA. METTL3 is a lentiviral vector constructed for METTL3 overexpression, and CTR as negative control of vector

As the only methyltransferase with catalyzing activity, METTL3 showed a remarkable high expression level in immature DPSCs. To investigate the biological effect of METTL3 inhibition in mature DPSCs, two independent lentiviral vectors were constructed to knockdown METTL3 expression. Both METTL3 shRNA-1 and shRNA-2 could inhibit its mRNA, protein expression in DPSCs (Fig. 3a, S2A), and so does lentiviral vector-overexpressing METTL3 (Fig. 3d, S2B). Representative images of cell morphology showed that shMETTL3-DPSCs exhibited elongate spindle shape and enlarged size compared with the control group (Fig. 3b). Also, β-galactosidase (β-gal) staining detected an increasing number of β-gal-positive cells after METTL3 inhibition which indicated DPSCs senescence (Fig. 3c, d). CCK8 assay showed no significant differences in cell viability in DPSCs (Fig. 3f) while flow cytometry demonstrated a significantly higher apoptosis rate after METTL3 knockdown (Fig. 3g). Additionally, phosphorylation of apoptotic-related p53 protein was upclined, and TUNEL assay showed increasing apoptosis by METTL3 knockdown (Fig. 3h, i). These data suggested that METTL3 inhibition in immature DPSCs induced cell senescence and apoptosis which could relate to the reduced self-renewal and regenerative potential.

### Bioinformatic analysis of METTL3 knockdown in DPSCs

**Fig. 4:**
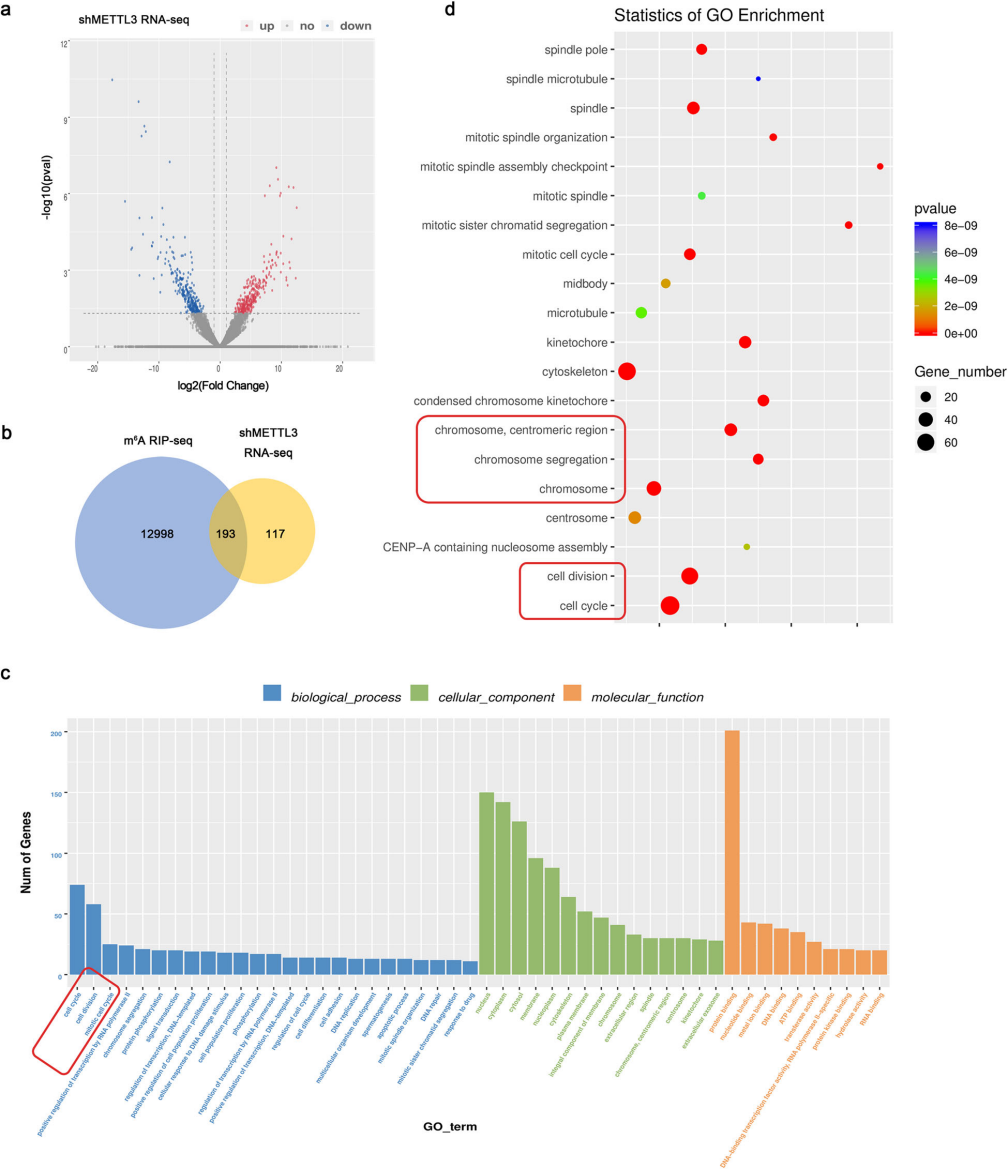
Bioinformatic analysis of METTL3 knockdown in DPSCs. **a** Volcano plots of differentially expressed genes (DGEs) in shCTR and shMETTL3-DPSCs (vertical lines represent > 2.0 fold change, horizontal lines for *p* values < 0.05). Red dots for upregulated and green for downregulated genes. **b** Venn diagram exhibited overlapped DEGs in shMETTL3-RNA-seq and m^6^A methylated transcripts in m^6^A RIP-seq. **c** Top terms identified by Gene Ontology enrichment analysis of overlapped genes including biological processes (BP), cellular components (CC), and molecular functions (MF). **d** Scatter plots of the top items analyzed in Gene Ontology enrichment

RNA-seq was carried out to screen the transcriptome profile of DPSCs transfected with two independent shMETTL3 vectors. A list of total 312 genes with GeneBank accession number from the National Center for Biotechnology Information (NCBI) database were identified as differentially expressed genes (DEGs) in DPSCs (*p* value < 0.05, FC > 2 or < − 2). Among these genes, 130 genes were upregulated while 182 ones were downregulated (Fig. 4a).

Bioinformatic analysis combing 13,191 m^6^A-methylated genes identified in m^6^A RIP-seq and 312 DEGs in shMETLL3 RNA-seq revealed that 216 genes were overlapped which might be regulated by METTL3-mediated m^6^A RNA methylation (Fig. 4b). Gene Ontology Consortium (GO) analysis was performed to functionally enrich the overlapped genes into specific biological contexts and summarize genes of related functions. For cell component (CC), genes were mainly enriched in the plasma membrane nucleus, while genes exhibited significant enrichments in protein binding for molecular function (MF) (Fig. 4c). Biological processes (BP) were essential to evaluate stem cell activities. Cell cycle, cell division, and positive regulation of transcription were the top 3 related processes which were related to the self-renewal and regenerative capacity of stem cells (Fig. 4c, d). In summary, the alternative gene expressions enriched in items as cell cycle, chromosome which indicated that cell cycle was the most relevant function resulted from METTL3 knockdown in DPSCs.

### Alteration of METTL3 disrupted cell cycle via PLK1 m^6^A modulation

**Fig. 5:**
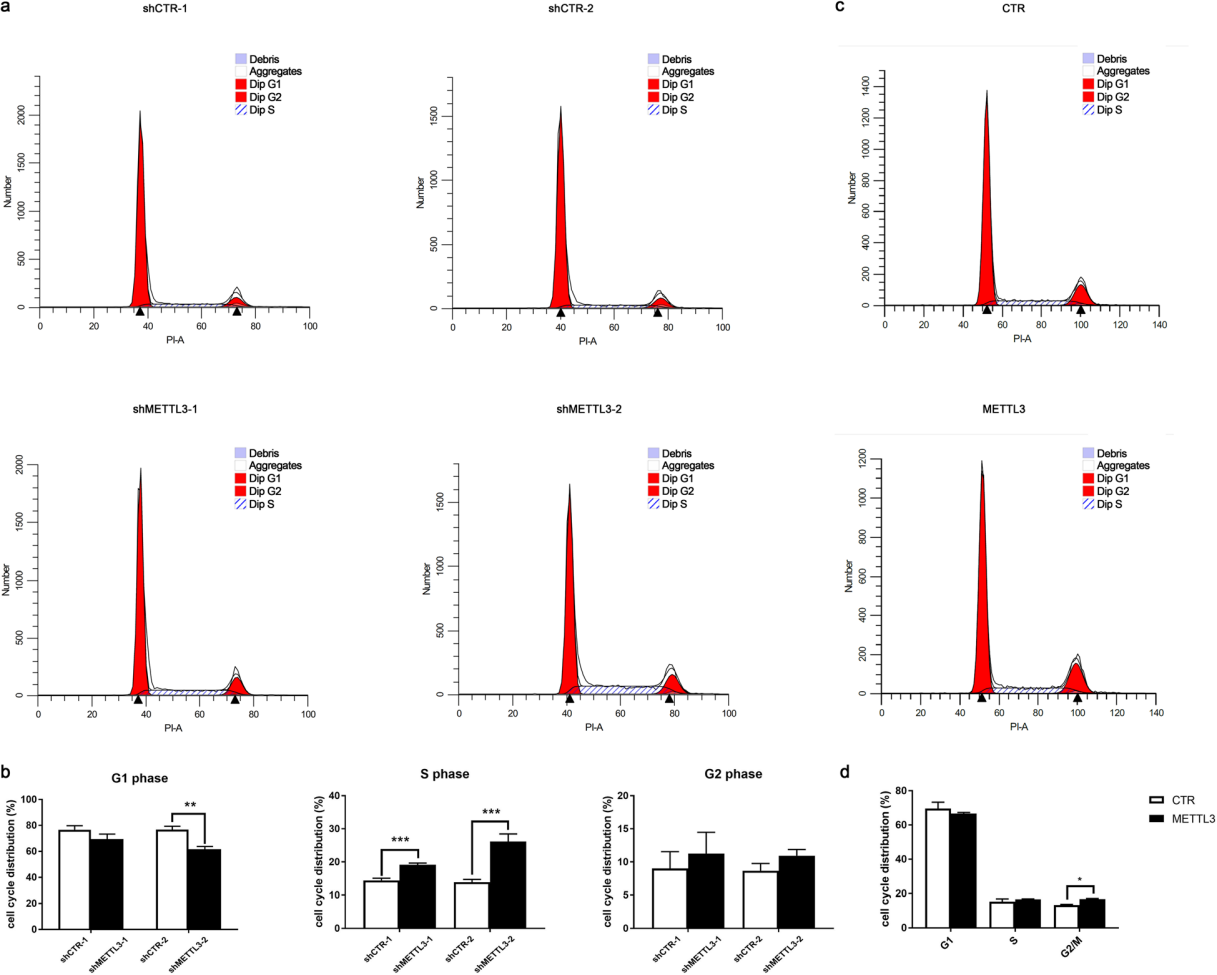
Alteration of METTL3 expression disrupted cell cycle progression of DPSCs. Flow cytometry detected the distribution of DPSC cell cycle phase after METTL3 knockdown by two independent METTL3-shRNA lentiviruses. **a** shCTR-1 and shMETTL3-1 treated DPSCs. **b** shCTR-2 and shMETTL3-2 treated DPSCs. **c** Cell cycle phase portions in METTL3-overexpressed DPSCs analyzed with flow cytometry. Data are represented as mean ± SD (*n* = 3), Student’s *t* test. *p < 0.05. **p < 0.01. ***p < 0.001

Flow cytometric analysis was performed to analyze the biological role of METTL3 in DPSCs cycle distribution. METTL3 knockdown by both shMETTL3 lentiviral vectors in DPSCs showed upregulated in the percentage of S phase while no significant difference was found in G2-M phase comparing to the negative control group which suggested METTL3 knockdown contributed to S phase cycle arrest in DPSCs (Fig. 5a, b). METLL3 overexpression led to different cycle distribution with a lightly upregulated in G2-M phase, and no variant in both G0-G1 and S phase (Fig. 5c, d).

**Fig. 6:**
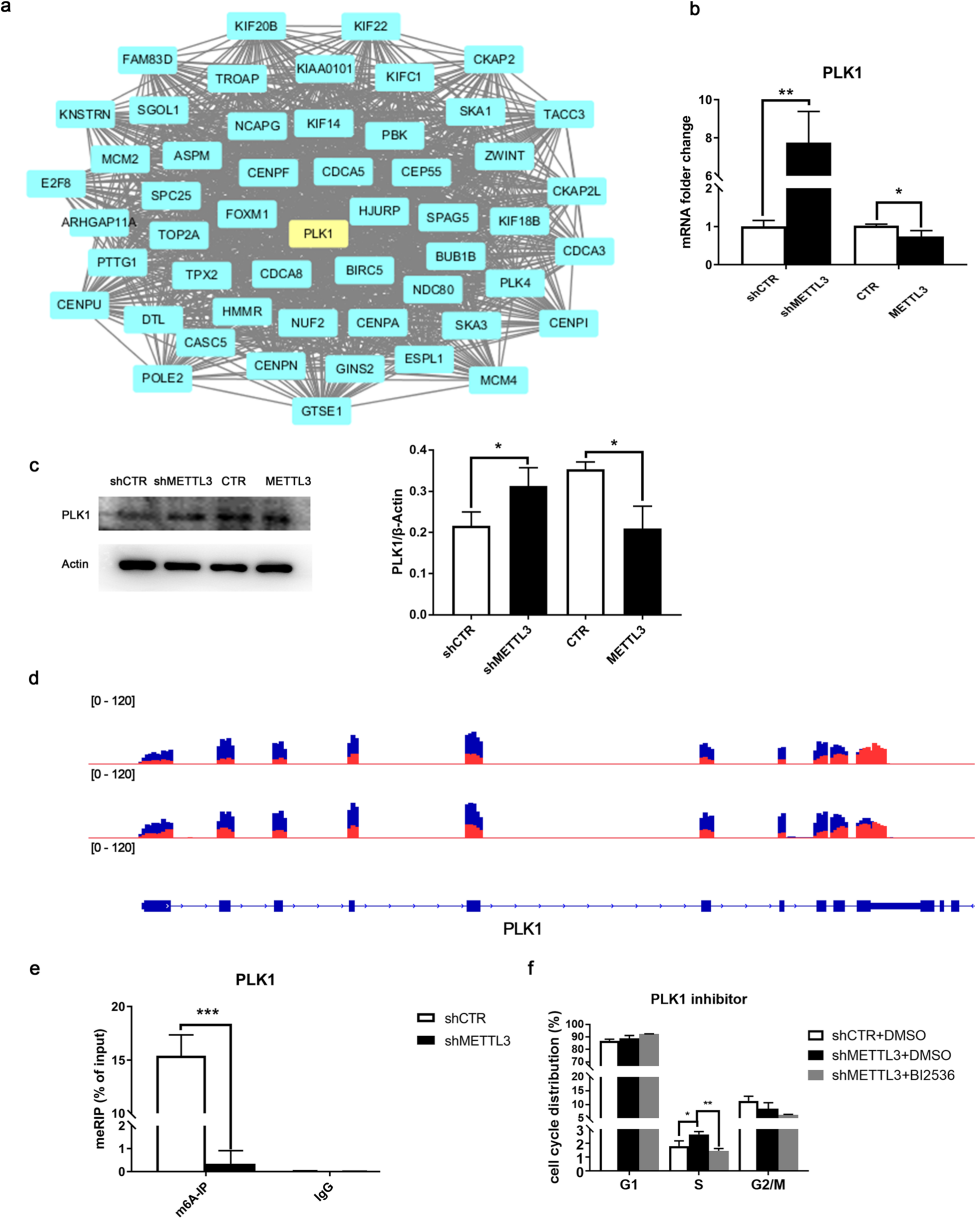
METTL3 regulated cell cycle via PLK1 m^6^A modulation. **a** Predicted protein-protein interaction (PPI) network of overlapping genes was assayed in the STRING database and displayed by Cytospcape which identified PLK1 as one of the key nodes in PPIs. **b** mRNA expression of PLK1 in DPSCs after METTL3 knockdown and overexpression detected by PCR. **c** Protein expression of PLK1 assayed by Western blot. **d** Visual data of highly enriched m^6^A peaks in PLK1 detected by m^6^A RIP-seq. **e** m^6^A RIP-qPCR demonstrated m^6^A-methylated level of PLK1 sharply decreased after METTL3 inhibition. **f** Flow cytometry analysis evaluated cell cycle portions in shMETTL3-DPSCs treated with 1 nM PLK1 inhibitor BI2536 or DMSO as negative control. Data are represented as mean ± SD (*n* = 3), Student’s *t* test. *p < 0.05. **p < 0.01. ***p < 0.001

To further explore the underlying mechanism of the impaired cell cycle regulated by METTL3, protein-protein interactions (PPIs) of DEGs were screened by the STRING database and network was then subjected to Cytoscape for further analysis (Fig. 6a). A critical cycle and mitosis modulator, Polo-like kinase 1 (PLK1), was identified as the central node of PPI network. qPCR and Western blot were used to confirm PLK1 expression after METTL3 alteration. mRNA and protein expression level of PLK1 were significantly upregulated after METTL3 knockdown while downregulated in METTL3 overexpression (Fig. 6b, c). Data visualization of m^6^A RIP-seq displayed the specific m^6^A peaks and distribution in PLK1 and m^6^A RIP-qPCR demonstrated m^6^A modification level fallen off a cliff after METTL3 inhibition in DPSCs (Fig. 6d, e). In addition, PLK1 inhibitor BI2536 partly rescued the S phase cycle arrest induced by METTL3 knockdown (Fig. 6f). These data indicated METTL3-meditated PLK1 m^6^A modification regulated the cell cycle progression of DPSCs.

## Discussion

As the most prevalent, reversible internal modification in nuclear RNAs, m^6^A modifications are involved in various biological processes including stem cell fate determination, embryonic development, and cell cycle control [25–27]. m^6^A methyltransferase complex and demethylases cooperated to influence different life stages of stem cells in post-translational levels [10]. DPSCs served as a promising cell source in endodontic regeneration and tissue engineering; however, its regenerative capacity is limited and reduced in a long lifespan. Our data first identified the binding area, motif, and distribution of m^6^A peaks in DPSCs by m^6^A RIP-seq. The expression level of m^6^A methyltransferases-METTL3 was relatively high in immature DPSCs which are known to possess superior regenerative potential. METTL3 inhibition in immature DPSCs induced cell senescence and apoptosis. A conjoint analysis of RNA-seq and m^6^A RIP-seq showed METTL3 knockdown mainly associated cell cycle and chromosome stability that tightly related to stem cell self-renewal and fate decision. Mechanistically, the alteration of METTL3 led to cell cycle arrest in DPSCs by regulating m^6^A modification of PLK1. Our data indicated that METTL3 may support sufficient regenerative potential in DPSCs by cell cycle control.

Over 80% of transcripts were methylated during development which occur to effect the self-renewal and pluripotency of stem cells [28, 29]. m^6^A modification can influence stability, altered splicing, exporting, translation of mRNA in a fine, and cell-specific manner. All m^6^A marks share the redundant consensus RRm6ACH ([G/A/U][G>A]m6AC[U>A>C]) motif which is enriched in 3′ and 5′ UTR of RNA transcripts especially long internal exons [30]. In our study, m^6^A RIP-seq revealed that in DPSCs, the consensus motif of m^6^A was GGACAG and mainly distributed in 3′ UTR, then 5′ UTR and the methylated level were higher in exons than introns which was in consistent with previous studies. The clustered m^6^A modifications in 3′ UTR around the stop codon influenced stability, localization, and translation of RNA while in 5′ UTR effected translation in a cap-independent manner [14, 31]. Methyltransferase complex is responsible to distinguish and target m^6^A sites, and METTL3 is the only catalytically active subunit which catalyzes the methylation of adenosines to form m^6^A modification [32]. METTL3 can independently read, regulate m^6^A marks near 3′ UTR of mRNA and promote translation of this specific mRNA [33]. Here, we identified METTL3 was the essential m^6^A-related gene differentially expressed in different stages of DPSCs which might relate to regenerative capacity alteration.

METTL3-mediated m^6^A methylation modified the transcripts in a cell-dependent manner which lead to divergent effects, somehow even conflicting [28]. METTL3 knockdown and m^6^A depletion could limit self-renewal and promote cell differentiation in epiblast stem cells while improving pluripotency, block regeneration in embryonic stem cells [13, 26, 34]. A recent study suggested METTL3 knockout accelerated mesenchymal stem cell senescence by modulating m^6^A modification of MIS12 which supported our data [35]. In our study, METTL3 inhibition in immature DPSCs promoted cell senescence and apoptosis which might further result in reduced regenerative potential in mature teeth. METTL3 depletion could make a contrary effect in apoptosis in different states. Some studies reported that knockdown METTL3 could rescue cell apoptosis induced by high glucose in human lens epithelial cells and also hypoxia/reoxygenation-treated cardiomyocytes [36, 37]. Meanwhile, METTL3 conditional knockout induced apoptosis of newborn granule cells which led to manifest cerebellar hypoplasia [38]. Furthermore, METTL3 inhibition promoted cell apoptosis by modulating m^6^A modification in multiple cancers [17, 39]. We found that knockdown METTL3 induced DPSC apoptosis along with p53 pathway activation which could impair stem cell self-renewal and fate decision.

Functional stem cell senescence and apoptosis are cooperated with proper cell cycle progress which make essential effect in cellular homeostasis. Prime stem cells usually stayed quiescence as a cell resource; progenitor cell expansion needs a high cycling rate or shorter time, while some differentiated cells withdraw from the cell cycle could still reenter under certain signals [40, 41]. Dynamic activation of the cell cycle was responsible for bone marrow stem cell self-renewal and hematopoietic stem cell maturation [42]. The transcriptome and epigenetic landscape alterations in cell cycle correspond with different status in development, maturation, and differentiation [41]. m^6^A modifications were reported to participate in stem cell self-renew and pluripotency by cell cycle control [29, 43, 44]. In cortical neurogenesis, the reduction of m^6^A modification resulted in a longer cell cycle which related to insufficient differentiation and delayed generation [29]. By bioinformatics analysis, we found inhibition of METTL3-meditated m^6^A mainly enriched in cycle-mediated factors and METTL3 alteration in DPSCs led to the cell cycle arrest in DPSCs. These findings were supported by recent studies which indicated a regulatory role of METTL3-meditated m^6^A methylation in cell cycle control. Methyltransferase complex consisted of METTL3, METTL14, and WTAP, inhibition of either could contribute to cell cycle arrest and disrupt the adipogenesis of adipocytes [45]. As an oncogene, METTL3 depletion could result in cell cycle arrest in acute myeloid leukemia and bladder cancer in a m^6^A-dependent manner [46, 47]. METTL3 was also reported to be a tumor suppressor by regulating cell cycle and self-renewal in renal cell carcinoma and glioblastoma [48, 49].

Cell cycle progress is controlled by robust cell cycle-related genes and checkpoints which were essential for self-renewal and homeostasis of stem cells. As a critical mitotic cell cycle regulator, PLK1 make effect on centrosome maturation, cytokinesis execution, bipolar spindle formation, and chromosome segregation. The expression of PLK1 was tightly associated with cell cycle stages that it initiated at a relatively low level and accumulated during the S phase, then reach a peak in the G2/M phase [50, 51]. PLK1 was widely studied as a target in cancer therapy while residued relative low expression level in normal cells. PLK1 overexpression in mammary epithelial and murine cells contributed to mitosis defection and aberration in chromosome segregation, cytokinesis which supported unscheduled PLK1 expression disrupted mitosis, and defected genomic stability [52, 53]. Consistent with this study, we discovered the expression of PLK1 in DPSCs and elevated PLK1 expression following METTL3 inhibition resulted in the S phase arrested which suggested a regulatory role of PLK1 in cell cycle control of DPSCs. PLK1 was reported to mediate G2 And S-phase expressed 1 (GTSE1) phosphorylation and p53 inactivation which led to G2 checkpoint recovery, DNA damage checkpoints termination, and chromosome instability [54]. In this study, our data showed that METTL3 regulated PLK1 expression in a m^6^A-dependent manner and participated in cell cycle control, which further effect DPSC senescence and apoptosis.

## Conclusions

Taken together, we demonstrated that m^6^A modification and METTL3 alteration participated in different stages of DPSCs which would relate to stem cell fate determination. Impaired METTL3 expression in DPSCs led to increasing cell senescence and apoptosis by interfering with the mitotic cell cycle in a m^6^A-dependent manner. Our results first uncovered the m^6^A methylated hallmarks in DPSCs and revealed the potential link between METTL3-mediated m^6^A modification and cell cycle control. Further researches are still needed to confirm these findings in vivo and enrich the RNA epigenetic mechanisms in pluripotency and self-renewal of DPSCs which would advance our understanding and serve new strategies in endodontic regeneration and tissue engineering.

## Supplementary Information

**Additional file 1: Supplementary Materials- Table 1. **The sequence of primers used in PCR.

**Additional file 2: Supplementary Figure S1.** The images of DPSCs under microscope.

**Additional file 3: Supplementary Figure S2.** Quantitative analysis of protein expression bands after METTL3 inhibition and expression.

## Data Availability

The datasets used and/or analyzed during the current study are available from the corresponding author on reasonable request.

## Abbreviations

DPSCs: Dental pulp stem cells
m^6^A: *N*^6^-methyladenosine
m^6^A RIP-seq: m^6^A immunoprecipitation with deep sequencing
METTL3: Methyltransferase like 3
PLK1: Polo-like kinase 1
METTL14: Methyltransferase like 14
WTAP: Wilms tumor 1-associated protein
FTO: Fat mass and obesity-associated protein
ALKBH5: alkB homolog 5
ALP: Alkaline phosphatase
ARS: Alizarin red staining
m^6^A RIP-Seq: m^6^A-methylated RNA immunoprecipitation sequencing
PCR: Real-time polymerase chain reaction
cDNA: Complementary DNA
β-gal: β-Galactosidase
GO: Gene Ontology
STRING: Retrieval of Interacting Genes
YTHDF: YTH domain-containing family protein
NCBI: National Center for Biotechnology Information
UTR: Untranslated regions
TSS: Transcription start sites
BP: Biological processes
CC: Cell component
MF: Molecular function
PPIs: Protein-protein interactions
GTSE1: G2 And S-phase expressed 1
