# Supplementary material for: METTL3-mediated m6A modification regulates cell cycle progression of dental pulp stem cells
Source: Stem Cell Res Ther. 2021 Mar 1;12:159. doi: 10.1186/s13287-021-02223-x (PMC7923612; doi:10.1186/s13287-021-02223-x)
Supplement: Supplementary file 1 — Additional file 1: Supplementary Materials- Table 1. The sequence of primers used in PCR. [file 13287_2021_2223_MOESM1_ESM.docx]

**Supplemental Materials- Table 1：The sequence of primers used in PCR**

| GAPDH | F | TCAACAGCGACACCCACTC |
| --- | --- | --- |
|  | R | GCTGTAGCCAAATTCGTTGTC |
| COL1 | F | GCTCGTGGAAATGATGGTGC |
|  | R | ACCCTGGGGACCTTCAGAG |
| ALP | F | CCAAAGGCTTCTTCTTGCTG |
|  | R | CCACCAAATGTGAAGACGTG |
| RUNX2 | F | TCGCCAGGCTTCATAGCAAA |
|  | R | GGCCTTGGGTAAGGCAGATT |
| BSP | F | AAGGACAAGGCTACGATGGC |
|  | R | CGGATGCAAAGCCAGAATGG |
| OCN | F | CATGAGAGCCCTCACACTCC |
|  | R | CTCCTGAAAGCCGATGTGGT |
| DSPP | F | ATATTGAGGGCTGGAATGGGGA |
|  | R | TTTGTGGCTCCAGCATTGTCA |
| CREB | F | AGAAGCGGAGTGTTGGTGAG |
|  | R | TCACTGACATCCTGCTTTACAA |
| LPL | F | CAAGAGTGAGTGAACAAC |
|  | R | AATTATGCTGAAGGACAAC |
| METTL3 | F | GAGGAGTGCATGAAAGCCAG |
|  | R | GGCCTCAGAATCCATGCAAG |
| METTL14 | F | GACGGGGACTTCATTCATGC |
|  | R | CCAGCCTGGTCGAATTGTAC |
| WTAP | F | ACGCAGGGAGAACATTCTTG |
|  | R | CACACTCGGCTGCTGAACT |
| FTO | F | AGACACCTGGTTTGGCGATA |
|  | R | CCAAGGTTCCTGTTGAGCAC |
| ALKBH5 | F | ACCCCATCCACATCTTCGAG |
|  | R | CTTGATGTCCTGAGGCCGTA |
| PLK1 | F | TGACTCAACACGCCTCATCC |
|  | R | AGGAGACTCAGGCGGTATGT |
