# Supplementary figures and images for: METTL3-mediated m6A modification regulates cell cycle progression of dental pulp stem cells
Source: Stem Cell Res Ther. 2021 Mar 1;12:159. doi: 10.1186/s13287-021-02223-x (PMC7923612; doi:10.1186/s13287-021-02223-x)

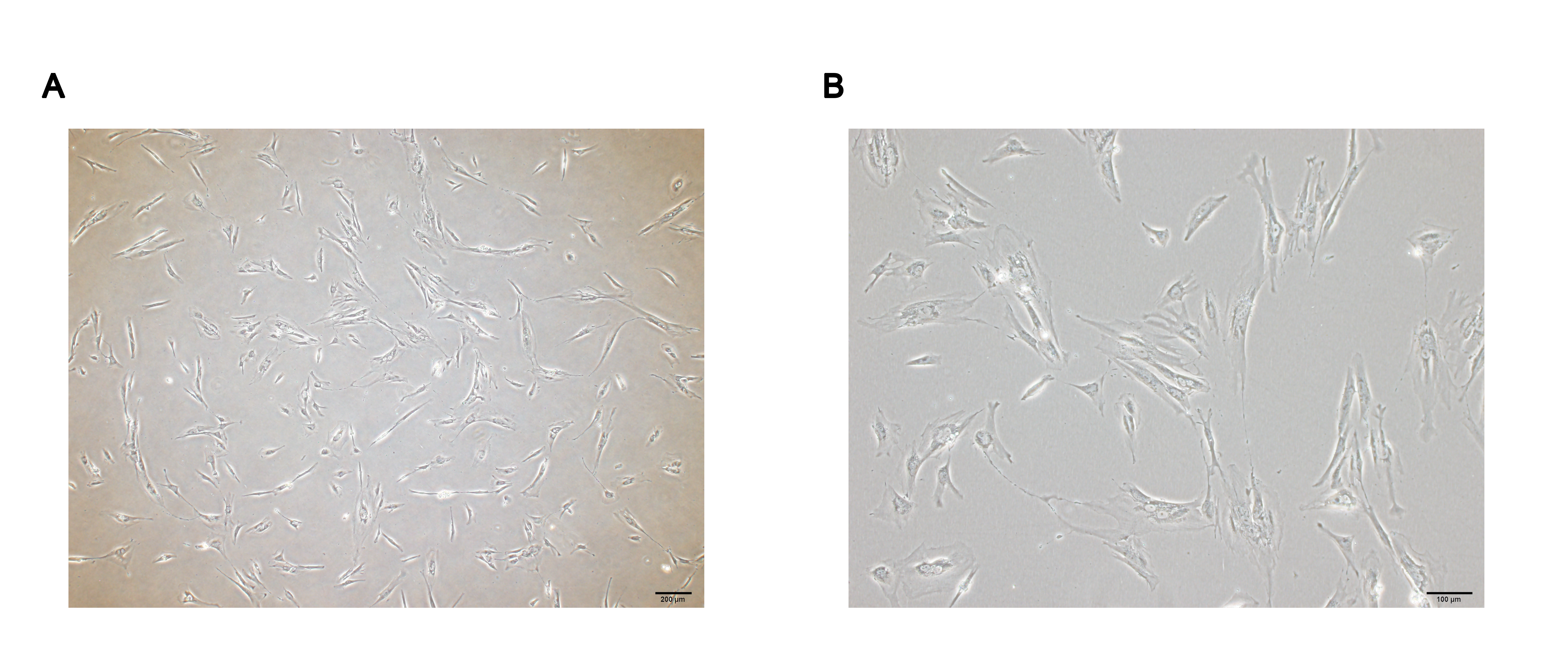

Supplement: Supplementary file 2 — Additional file 2: Supplementary Figure S1. The images of DPSCs under microscope. [file 13287_2021_2223_MOESM2_ESM.tif]

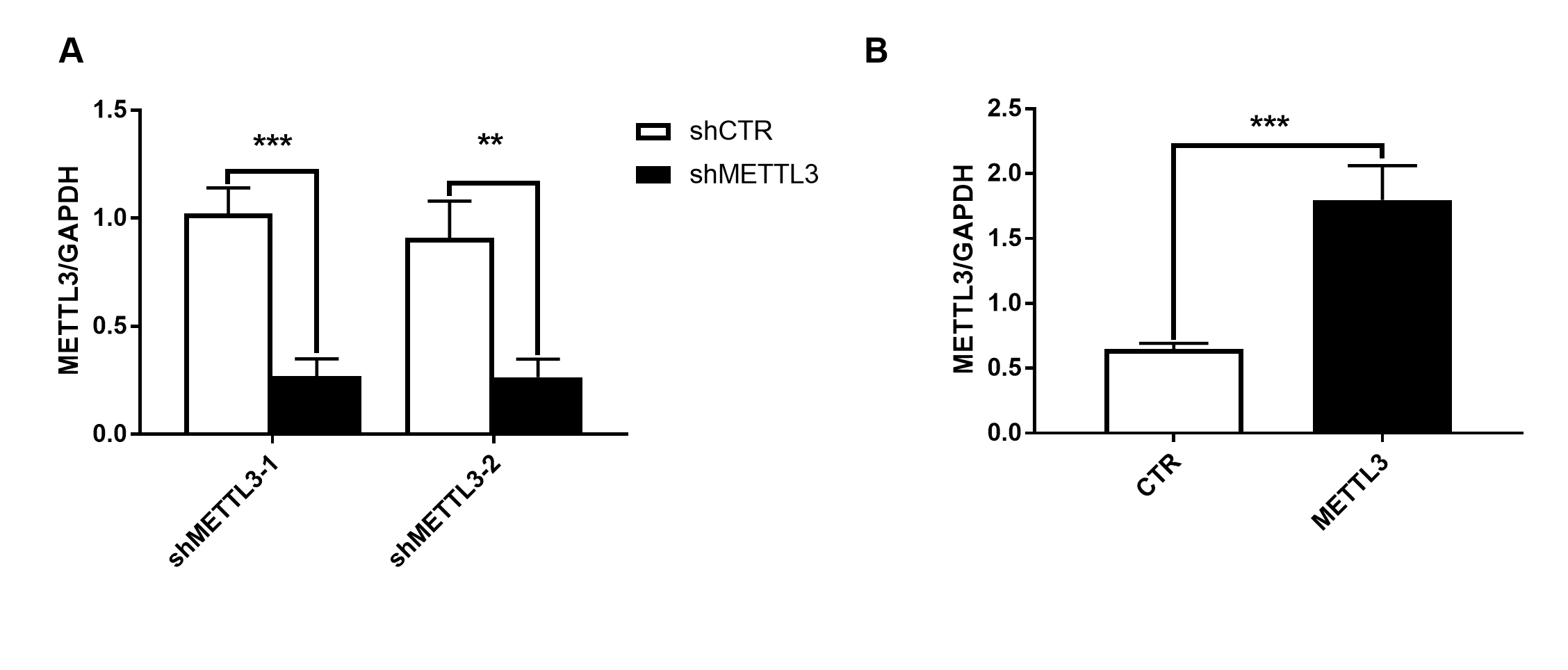

Supplement: Supplementary file 3 — Additional file 3: Supplementary Figure S2. Quantitative analysis of protein expression bands after METTL3 inhibition and expression. [file 13287_2021_2223_MOESM3_ESM.tif]
